# Highly accelerated single breath-hold non-contrast thoracic MRA: evaluation in a clinical population

**DOI:** 10.1186/1532-429X-15-S1-E84

**Published:** 2013-01-30

**Authors:** Ruth P Lim, Priscilla A Winchester, Mary T Bruno, Jian Xu, Pippa Storey, KellyAnne McGorty, Daniel K Sodickson, Monvadi B Srichai

**Affiliations:** 1 Radiology, Austin Health, Heidelberg, VIC, Australia; 2 Radiology, NYU Langone Medical Center, New York, NY, USA; 3 Research and Development, Siemens Medical Solutions, New York, NY, USA

## Background

Gadolinium enhanced MRA (Gd-MRA) is commonly used in clinical practice for evaluation of the thoracic aorta. Electrocardiographic (ECG) gating is required for accurate aortic root assessment, but decreases scanning efficiency. We evaluate the performance of a highly accelerated, breath-hold 3D ECG-gated non-contrast enhanced steady state free precession MRA technique (NC-MRA) in a clinical population, compared with ECG-gated Gd-MRA.

## Methods

30 patients (22 male, mean age 53.4 years) with known or suspected thoracic aortic pathology were imaged with NC-MRA followed by Gd-MRA at 1.5T following informed consent. Images were anonymized and reviewed by 2 readers for aortic pathology. Diagnostic confidence, image quality and artifacts were evaluated on a 5-point Likert scale (1=worst, 5=best), with image quality and artifacts evaluated segmentally in 10 vascular segments, including the sinuses of Valsalva and coronary artery origins. Aortic dimensions were measured in 7 aortic segments. Diagnostic confidence, image quality and artefact scores were evaluated with the Wilcoxon signed rank test. Paired Student t-test and Bland-Altman analysis were used for comparison of aortic dimensions.

## Results

**Figure 1:**
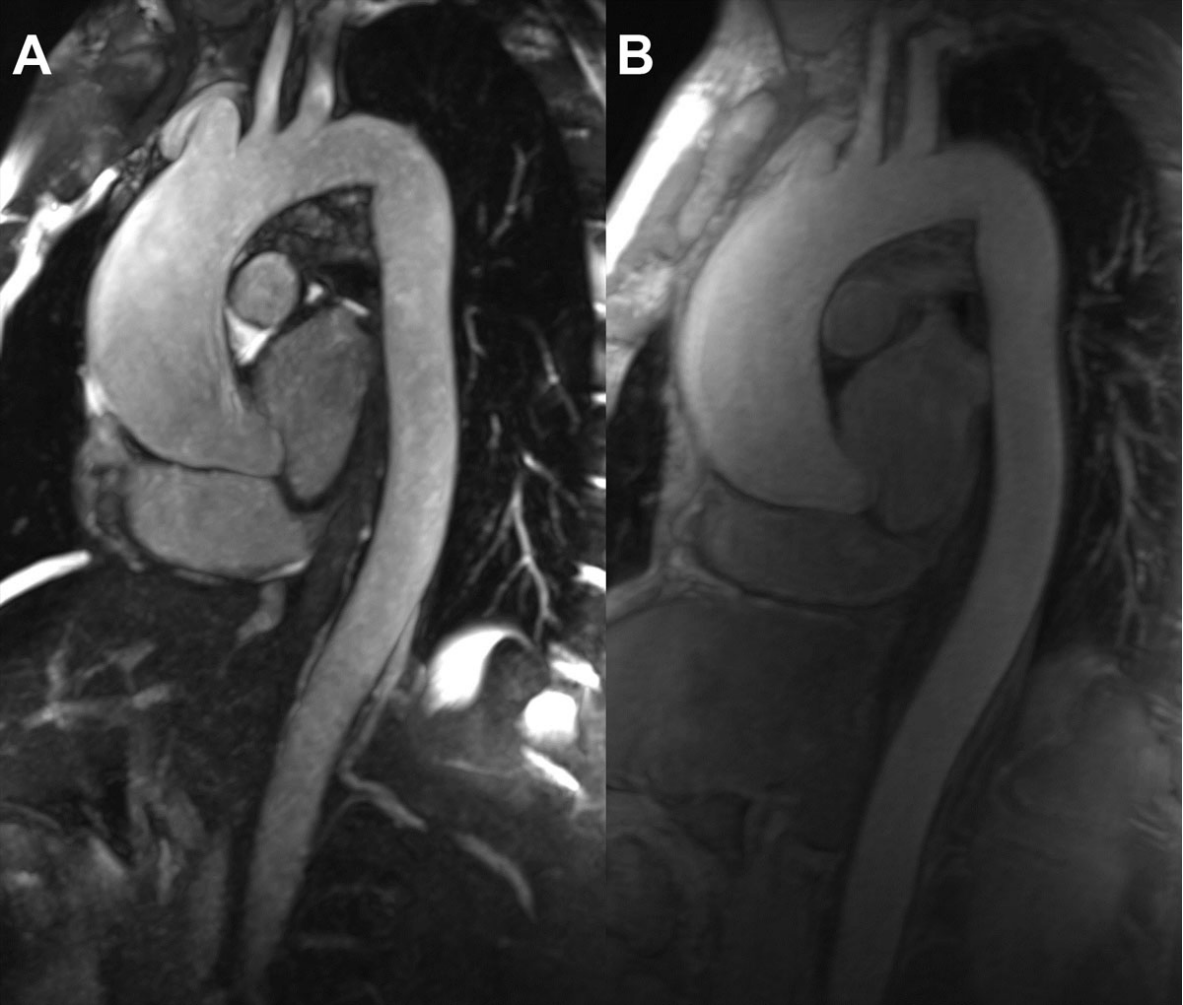
A) Non-contrast and B) gadolinium-enhanced MRA thin MIP images of a 42-year-old male with history of bicuspid aortic valve. The ascending aorta is aneurysmal, measuring up to 4.6cm, and is well depicted with the non-contrast MRA technique.

All patients successfully completed NC-MRA and Gd-MRA (Figure 1). NC-MRA vascular pathology findings were concordant with Gd-MRA in 29/30 (96.7%) and 28/30 (93.3%) of patients for Readers 1 and 2 respectively with high diagnostic confidence (mean 4.35±0.77), not significantly different from Gd-MRA (4.38±0.64), p=0.74. Image quality and artefact scores were comparable with Gd-MRA in the majority of vascular segments. Differences were observed at the ascending aorta, where NC-MRA image quality (3.80±0.88) was inferior to Gd-MRA (4.13±0.73), and at the coronary artery origins, where NC-MRA was considered superior for the left main (3.38±1.47 versus Gd-MRA 2.78±1.21) and the right coronary (3.55±1.40 versus 2.32±1.16) arteries, p<0.05 for both comparisons. Aortic dimensions were comparable, with only one significant difference observed at the ascending aorta, where mean NC-MRA dimension (4.05±0.76 cm) was less than 1 mm smaller than Gd-MRA (4.12±0.70), p=0.043.

## Conclusions

Breath-hold non-contrast enhanced MRA of the thoracic aorta yields good image quality, comparable to ECG-gated gadolinium-enhanced MRA, with high accuracy for aortic dimensions and pathology. It can be considered an alternative to gadolinium-enhanced MRA in patients with relative contra-indications to gadolinium contrast or problematic venous access.

## Funding

This project was supported by grant number K12HS019473 from the Agency for Healthcare Research and Quality. The content is solely the responsibility of the authors and does not necessarily represent the official views of the Agency for Healthcare Research and Quality. D.K.S. and J.X. receive grant support from National Institutes of Health R01-DK069373. J.X. is an employee of Siemens Medical Solutions.

